# Fertility sparing surgery on placenta invasion anomalies and placenta previa

**Published:** 2012-05

**Authors:** Ateş Karateke, Mehmet Küçükbaş, Hamdullah Sozen, Ahmed Namazov, Seda Çakır, Yesim Akdemir

**Affiliations:** 1 *Zeynep Kamil Training and Research Hospital, Uskudar, İstanbul, Turkey.*; 2 *Sakarya Education and Training Hospital, Sakarya, Turkey.*

**Keywords:** *Placenta accreta*, *Bakri balloon tamponade*, *Internal iliac artery ligation*

## Abstract

**Background**: Abnormal placental invasion has increased parallel with persistent rise in Caesarean delivery. Management relies on accurate diagnosis and delivery should be planned at an institution with appropriate expertise and resources for managing this condition.

**Case:** We present a case of a placenta invasion anomaly which is the major risk factors of peripartum deaths. In this case we try to explain our approach which reduces unnecessary hysterectomy rates.

**Conclusion:** In order to avoid postpartum hemorrhage and hysterectomy protocols, our approach which consists bilateral hypogastric arterial ligation, Bakri balloon tamponade and ıf necessary methotrexate therapy can be applied succesfully.

## Introduction

Placenta accreta may be defined as an abnormal adherence of the placenta to the uterine wall. There is a deficiency of the decidua and there is myometrial invasion by chorionic villi (1). Abnormal placental adherence has been classified into accreta; increta and percreta based on dept of invasion into the myometrium. Major risk factors are placenta previa and prior caesarean delivery (2). The incidence of placenta accreta is increasing parallel with the increase in caesarean delivery.

Placenta accreta has been associated with postpartum hemorrhage and maternal morbidity and mortality. Post-partum hemorrhage is a major complication of the delivery, responsible for a quarter of maternal deaths worldwide (3) and a major cause of maternal mortality in the UK (4).

The traditional approach for third trimester hemorrhage due to abnormal placental attachment is Caesarean delivery. Caesarean is performed with uterine Kehr incision and after baby born placenta is removed from the uterine cavity. Sometimes this approach will cause serious hemorrhage and decrease the level of coagulation factor.

In our case prophylactic artery ligation technique and Bakri tamponade is preferred to avoid massive postpartum hemorrhage. Before the removal of the placenta, bilateral internal iliac artery ligation was performed. After that the uterine wall was evaluated and there were no defect on uterine serosa, Bakri tamponade was placed in the uterine cavity. Patient was discharged 3 days later with no complication of hemorrhage and abnormal placental invasion.

## Case Report

**Figure 1:**
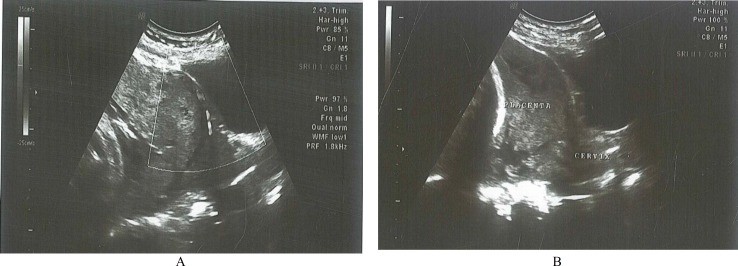
Turbulent internal flow and thinnig of the myometrium

A healthy 36 years old woman who had pregnancy complicated by placenta previa and placenta accreta was referred to our hospital. She had a history of twice caesarean section which one of them was due to placenta previa. In ultrasonographic examination the internal cervical os was covered completely by placenta. In addition the sonographic examination of placenta revealed multiple hipoechoic spaces (lacunae), turbulent internal flow and thinnig of the myometrium [Figure 1 (A, B)]. These findings were interpreted in favor of total placenta previa and abnormal placental invasion.

The patient was scheduled for an elective Caesarean section at 38 weeks. The patient was informed the type of operation and possible complications which is related with procedure. Preoperatively hemoglobin was calculated as 10.2 g/dl. All the coagulation profiles were found within normal range. Against the risk of bleeding and disseminated intravascular coagulation, four unit erythrocyte, eight unit platelet and eight unit plasma suspensions were prepared.

In the operating room the abdomen was penetrated by vertical incision. For the uterus high vertical incision was preferred instead of uterus Kehr incision in order to avoid bleeding of placenta. Subsequent to the removal of fetus; we waited 15 minutes to see normal placental seperation.At the end of this time normal separation could not be seen. Gentle traction was applied to remove the placenta but it could not be extracted from the uterine wall. 

By this step preoperational diagnosis of placental invasion anomaly was confirmed surgically. Retaining the placenta within the uterus, retroperiton was dissected in order to visualize the retroperitoneal vessels. External iliac and internal iliac artery was observed in the retroperitoneal area. Bilateral hypogastric artery ligation was done at 5 cm distal of arteria iliaca communis bifurcation. Following the ligation, placenta was removed from uterine cavity by strong traction. Minimal placental tissues were cut with metzenbaum. 

The myometrium was invaded by the placental tissue. The uterine serosa was intact. These findings confirmed the diagnosis of placenta increta. Bleeding in the cervical region was observed and a Bakri balloon was placed into the uterine cavity. The uterine incision was separately sutured before filling balloon with saline solution. The tube was inflated with 500cc of hot saline solution. Bakri balloon stopped the small bleedings from the placental bed. Uterine vertical incision were closed by using interrupted absorbable sutures and continuous monoflament delayed absorbable suture was used to close the fascia. The skin of the abdomen was closed separately with non-absorbable sutures. The patient was not observed to have an extra bleeding. 

After 24 hours from the operation, 200cc of the tube was taken out and bleeding control on the patient showed no bleeding. After 26 hours, the remaining amount of 300cc was also taken out and the tube was displaced from the patient. Antibiotics were administered at duration of balloon usage to reduce the risk of iatrogenic infection caused by contamination of the uterine environment by the balloon from the vaginal environment. 

The hematocrit level of the patient was measured 30.2 before the operation and 26.8 after the operation. First 24 hours from the operation the distended uterus can cause discomfort and pelvic pain. In this period 50 mg Pethidin (Aldolan, Liba) was administered every 6 hours and Diclofenac (Diclomec, Abdi Ibrahim) sodium was applied two times. At 48 hours after the operation, the patient had no additional bleeding. 

Transvaginally ultrasonography showed no placental residual mass in the uterus. Therefor we did not perform methotrexate therapy. The patient was discharged on the 3rd postoperative day. Patient was examined every 7 days sonographically in the puerperium period. In this period no complication was seen. Her beta human chorionic gonadotropin (-hCG) level was 5mUI/mL in the 40th postpartum day.

## Discussion

Obstetric hemorrhage is the leading cause of the maternal mortality related to pregnancy (5). Abnormal placentation has emerged over uterine atony as the leading indication for peripartum hysterectomy (6). All the forms of abnormal placentation are associated with massive hemorrhage, which may lead to hypovolemic shock or a disseminated intravascular coagulopathy at delivery, and this will increase maternal morbidity and mortality.

Different treatment modalities are applied for peripartum hemorrhage in abnormal placental invasion. Uterine curettage, tamponade devices, peripartum hysterectomies, uterine artery ligation, hypogastric artery ligation, and hypogastric artery ballooning are the examples for the treatment. Hypogastric artery ballooning becomes very popular worldwide. 

However some studies have shown conflicting results concerning the efficacy of prophylactic hypogastric artery ballooning, some have reported satisfactory outcomes for decreasing the mean blood loss and transfusion requirement (7) and others have reported no benefit (8). The investigators, who failed to find a benefit with the endovascular occlusion, suggested that the mean blood loss did not decrease because of the rich collateral blood supply to the uterus. 

During the caesarean-sections of patients with invasion anomalies such as placenta accreta, increta, and percreta we observed very serious bleeding subsequent to the removal of placenta in our clinic. Some cases with heavy bleeding requiring peripartum hysterectomy. For cases with such invasion anomalies, we recommend penetrating into abdomen by vertical incision, applying the uterus high incision as far as possible from the placenta and taking out the fetus by this way. The purpose of this is to avoid harming the placenta and prevent the initiation of bleeding at placenta. Therefore, after the removal of fetus, we waited 15 minutes to see normal placental separation. In case that spontaneous placental separation cannot be seen, no efforts should be done to remove the placenta which will cause bleeding. First, bilateral hypogastric artery should be tied in order to mitigate the bleeding after removing the placenta. This process will reduce bleeding and allow the surgeon to properly manage the bleeding control of the patient. 

Disseminated intravascular coagulation may also be avoided by this way. After bilateral hypogastric artery ligation, the placenta should be removed. If necessary, placenta should be cut from the myometrium. After removing the placenta, the uterus wall should be evaluated. Provided that the uterus serosa has no defect, even when small placental residues exist in the uterus wall; the application of Bakri balloon to such patients will eliminate the need to do hysterectomy and prevent a possible life-threatening bleeding of the patient. By this method, the uterus and fertility of the patient will be protected. If hysterectomy will be applied, the approach should be, unlike the classical approach, visualizing the ureter and conducting type 2 hysterectomy. By this way, the probability of bleeding can be reduced by wide excision of the parametriums. If Bakri tube to be used, the Bakri tube balloon should be placed in the cervix and inflated 500cc. Bakri tube can make a pressure on the blood vessel which is more than the pressure within the vessel. The blood will clot and form a permanent seal if Bakri tube is applied long enough (9). 

24 hours after the placement, the tube should be emptied 200cc and bleeding should be controlled (10). If no bleeding exists, the tube should be taken out fully after 26 hours. Otherwise, tube should be inflated 200cc and taken out at the 48th hour. After the Bakri balloon extracted from uterus, ultrasonography was done in order to understand whether there was residual placental tissue or not? If placental remnant tissue was seen, methotrexate therapy can be administered. In our case no extra mass was seen in uterus, so we did not need methotrexate therapy. By this method, it becomes possible to prevent a severe bleeding in the patient.

Recently, uterine tamponade using intrauterine balloons become popular and appears to be an effective tool in the management of postpartum hemorrhage (11). The insufficient bleeding control is the most common complication after insertion of intrauterine balloon. In our approach, life threatening uterine bleeding after insertion of Bakri balloon was prevented by using bilateral hypogastric artery ligation which was done before Bakri balloon insertion and placenta removal.

Another method which became popular to prevent postpartum hemorrhage in case of placenta adhesion anomaly is conservative approach. At this method, placenta is retained in uterus and methotrexate therapy is administered in postoperative period. A review which investigated 48 articles reported that postpartum hemorrhage was common complication with a rate of 35/100 and 15% of women had to be treated by hysterectomy due to abnormal bleeding.18% of patient suffered puerperal infection after conservative treatment and 18% of them were hysterectomized because of the infections (12).

We think that high complication rates at conservative management is a result of high amount of retained placenta. But in our approach we try to retain placenta volume as low as we can. Especially at the centers where arterial balloon tamponade radiologically cannot be applied, our treatment model is an effective alternative that can be applied easily. 

## Conclusion

As a conclusion, in order to avoid postpartum hemorrhage and hysterectomy protocols, our approach which consists bilateral hypogastric arterial ligation, Bakri balloon tamponade and ıf necessary methotrexate therapy can be applied succesfully.

## References

[1] Lax A, Prince MR, Mennitt KW, Schwebach JR, Budorick NE (2007). The value of specific MRI features in the evaluation of suspected placentaL invasion. Magn Reson Imaging.

[2] Oyelese Y, Smulian JC (2006). Placenta previa, placenta accreta, and vasa previa. Obstet Gynecol.

[3] World Health Organization (WHO) (2005). Attending to 136 MillionBirths, Every Year: Make Every Mother and Child Count: The World Report 2005.

[4] Crowhurst JA, Plaat F (1999). Why mothers die- Report on confidential enquiries into maternal deaths in the United Kingdom 1994–96. Anaesthesia.

[5] Berg CJ, Atrash HK, Koonin LM, Tucker M (1996). Pregnancy-related mortality in the United States, 1987-1990. Obstet Gynecol.

[6] Stanco LM, Schrimmer DB, Paul RH, Mishell DR Jr (1993). Emergency peripartum hysterectomy and associated risk factors. Am J Obstet Gynecol.

[7] Kidney DD, Nguyen AM, Ahdoot D, Bickmore D, Deutsch LS, MajorsC (2001). Prophylactic perioperative hypogastric artery balloon occlusion abnormal placentation. AJR Am J Roentgenol.

[8] Greenberg JI, Suliman A, Iranpour P, Angle N (2007). Prophylactic balloon occlusion of the internal iliac arteries to treat abnormal placentation: a cautionary case. Am J Obstet Gynecol.

[9] Vitthala S, Tsoumpou I, Anjum Z (2009). , Aziz N Use of Bakri balloon in post-partum haemorrhage: A series of 15 cases Australian and New Zealand. J Obstet Gynaecol.

[10] Bakri YN, Amri A, Abdul Jabbar F (2001). Tamponade-balloon for obstetrical bleeding. Int J Gynaecol Obstet.

[11] Georgiou C (2009). Balloon tamponade in the management of postpartum haemorrhage: a Review. BJOG.

[12] Timmermans S, van Hof AC, Duvekot JJ (2007). Conservative management of abnormally invasive placentation. Obstet Gynecol Surv.

